# Association between neural prosody discrimination and language abilities in toddlers: a functional near-infrared spectroscopy study

**DOI:** 10.1186/s12887-024-04889-7

**Published:** 2024-07-12

**Authors:** YanRu Guo, YanWei Li, FuLin Liu, HuanXi Lin, YuYing Sun, JiaLin Zhang, Qin Hong, MengMeng Yao, Xia Chi

**Affiliations:** 1 Children’s Healthcare Department, Women’s Hospital of Nanjing Medical University (Nanjing Women and Children’s Healthcare Hospital), Nanjing, China; 2 College of Early Childhood Education, Nanjing Xiaozhuang University, Nanjing, China; 3 Southeast University, Nanjing, China; 4 State Key Laboratory of Reproductive Medicine and Offspring Health, Nanjing, China

**Keywords:** Preterm toddlers, Language delay, Functional near-infrared spectroscopy, Neural prosody discrimination, Prediction model

## Abstract

**Background:**

Language delay affects near- and long-term social communication and learning in toddlers, and, an increasing number of experts pay attention to it. The development of prosody discrimination is one of the earliest stages of language development in which key skills for later stages are mastered. Therefore, analyzing the relationship between brain discrimination of speech prosody and language abilities may provide an objective basis for the diagnosis and intervention of language delay.

**Methods:**

In this study, all cases(*n* = 241) were enrolled from a tertiary women’s hospital, from 2021 to 2022. We used functional near-infrared spectroscopy (fNIRS) to assess children’s neural prosody discrimination abilities, and a Chinese communicative development inventory (CCDI) were used to evaluate their language abilities.

**Results:**

Ninety-eight full-term and 108 preterm toddlers were included in the final analysis in *phase I* and *II* studies, respectively. The total CCDI screening abnormality rate was 9.2% for full-term and 34.3% for preterm toddlers. Full-term toddlers showed prosody discrimination ability in all channels except channel 5, while preterm toddlers showed prosody discrimination ability in channel 6 only. Multifactorial logistic regression analyses showed that prosody discrimination of the right angular gyrus (channel 3) had a statistically significant effect on language delay (odd ratio = 0.301, *P* < 0.05) in full-term toddlers. Random forest (RF) regression model presented that prosody discrimination reflected by channels and brain regions based on fNIRS data was an important parameter for predicting language delay in preterm toddlers, among which the prosody discrimination reflected by the right angular gyrus (channel 4) was the most important parameter. The area under the model Receiver operating characteristic (ROC) curve was 0.687.

**Conclusions:**

Neural prosody discrimination ability is positively associated with language development, assessment of brain prosody discrimination abilities through fNIRS could be used as an objective indicator for early identification of children with language delay in the future clinical application.

**Supplementary Information:**

The online version contains supplementary material available at 10.1186/s12887-024-04889-7.

## Background

Language delay in children is a phenomenon in which the language development of children lags behind their typically developing peers. The incidence of language delay is as high as 13.5%～17.5% in children aged 1.5～3 years [1, 2]. Delayed language development not only seriously affects children’s language comprehension and expression, but also often leads to reading and spelling difficulties, interpersonal abnormalities, and emotional and behavioral abnormalities, which can affect academic, occupational, and social development [3].

The problem was often overlooked in the past, because about 46% of children with language delays catch up with their peers by the age of 3～4 years, especially for children born full-term or low risk [4]. In fact, about 16% of children with early language delays will still have persistent language problems. A cohort study has found that some children with delayed language development catch up with children with normal language development, but most of these children continue to have learning difficulties related to narrative and reading when they reach the upper elementary grades [5].

Preterm birth are those that occur at less than 37 weeks’ gestational age (GA) [6]. Preterm birth is one of the important risk factors for language delay [7, 8]. Early detection of preterm infants with language delay allows for early intervention for better health, academic, and social outcomes before language delay causes subsequent problems. A previous study showed that about 25 ~ 30% of preterm infants aged 2.5 ~ 3.5 years have language delays and still have persistent comprehension difficulties at age 4 years [9]. In addition, a study of children and adolescents aged 8 ~ 9 years suggested that language development problems may not disappear with age [10].

Therefore, early screening for language delay in preterm infants is particularly important. The tools used by pediatricians to assess children’s language development in clinical settings include traditional methods such as language tests, observations, and questionnaires, all of which have their limitations and are unable to completely avoid subjectivity and bias in data collection, and may result in misdiagnosis or underdiagnosis [11]. Prosody is the basic information of language cognitive processing, which consists of the features of spoken language such as tone, pitch, volume, speech speed, stress and pause [12]. Prosody discrimination involves the brain’s ability to perceive and process prosodic elements within language [13]. The development of prosody discrimination is one of the earliest stages of language development in which key skills for later stages are mastered [14]. Therefore, analyzing the relationship between brain discrimination of speech prosody and language abilities, may provide an objective basis for the diagnosis and intervention of language delay.

Functional near-infrared spectroscopy (fNIRS) is a relatively new technique [15, 16]. It is used to measure and analyze the optical signals between the skull and the cerebral cortex, and to obtain the cerebral cortex’s oxygenated hemoglobin (HbO), deoxy-hemoglobin (HbR) concentrations at different points in time, which in turn reflects the activity of the cerebral cortex regions. With the advantages of noiseless, high anti-motion interference ability, relatively high spatial resolution, and safety, fNIRS provides a new approach for the study of brain function development (including brain prosody discrimination), diagnosis of brain diseases and brain neural mechanisms in children [17]. Previous studies applying fNIRS in the field of language delay show that fNIRS has clinical applications that may lead to improved early and differential diagnosis, increase our understanding of response to treatment, improve neuro prosthetic functioning, and advance neurofeedback [18].

Language delay affects near- and long-term social communication and learning in preterm and term infants, and an increasing number of experts pay attention to it. We investigated the associations between discrimination of speech prosody and language abilities in toddlers who were born full-term and preterm. We used fNIRS to assess children’s prosody discrimination abilities as it may provide an early warning of language delay in children.

## Methods

### Participants

All cases (*n* = 241) were enrolled from Children’s Healthcare Department in Women’s Hospital of Nanjing Medical University, from 2021 to 2022.Toddlers aged 1-year-old (12 months ± 1 month) and 2-year-old (24 months ± 1 month) were included, we use corrected age in preterm born children while use actual age in full-term born children as refer to previous research, for neuropsychological development in preterm children was adjusted for age up to 24 months [5].This study procedures were approved by the Medical Ethics Committee of the Women’s Hospital of Nanjing Medical University [No.2020(KY-058058)]. This two-phase study was designed to identify the associations between discrimination of speech prosody and language abilities and to determine risk factors for language delay. Inclusion criteria: normal hearing, both parents speak Chinese as their mother tongue, no known history of mental illness in parents and family. Exclusion criteria: combined history of serious complications that can cause severe brain damage after birth, chromosome abnormality or congenital abnormality, serious congenital malformation, genetic and metabolic diseases, serious audio-visual impairment, cerebral palsy, or who had a history of otitis media within 6 months.

**Fig. 1:**
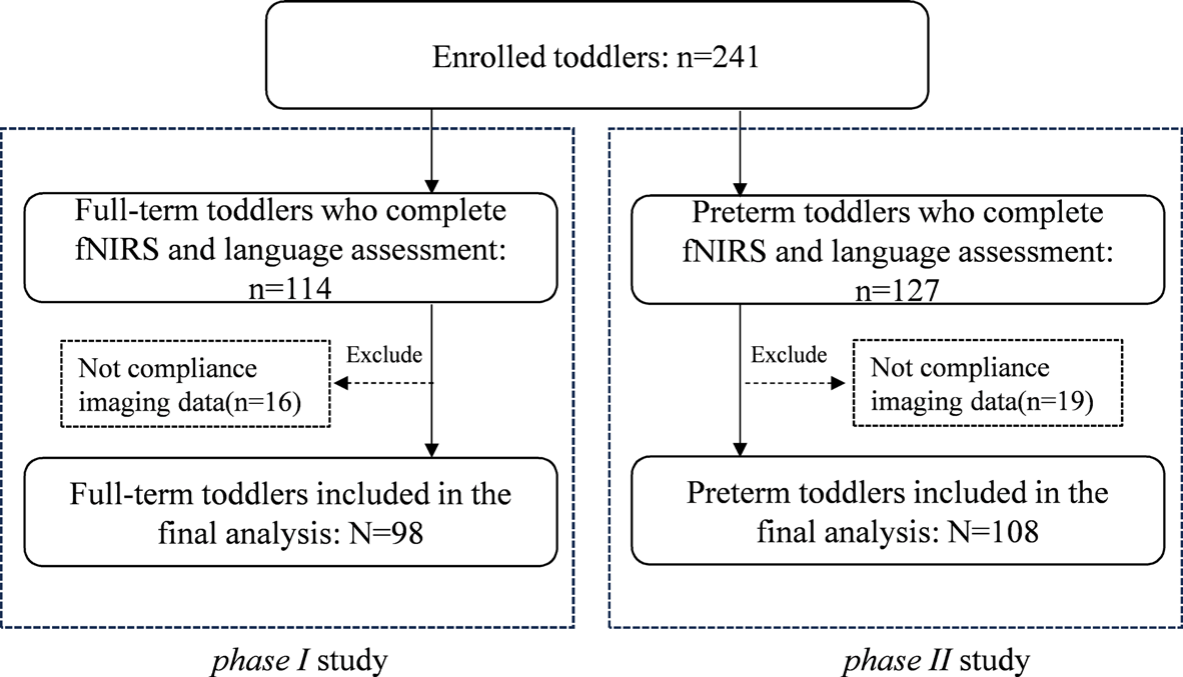
Flow chart of the population included in our final analysis

A total of 241 toddlers were recruited in this study in two phases, (1) the *phase Ι* study collected 114 children’s fNIRS and language assessment data to evaluate associations between discrimination of speech prosody and language abilities. Of them, 98 subjects had compliance imaging data and 16 toddlers were excluded because they had no compliance imaging data. (2) The *phase ΙΙ* study collected 127 children’s fNIRS and language assessment data. Of them, 108 subjects had compliance imaging data and 19 toddlers were excluded because of they had no compliance imaging data. The final samples included in the analysis were 98 in *phase I* study and 108 in *phase II* study (Fig. 1).

### Language measure

Language abilities were evaluated through the CCDI, a Chinese version of MacArthur-Bates Communicative Development Inventories [19], which includes Words and Gesture long-Form (6 dimensions, including early gesture, late gesture, total gesture, phrase comprehension, vocabulary comprehension, vocabulary expression) for 1-year-old toddlers, and Words and Sentences long-Form (2 dimensions, including Vocabulary expression, sentence complexity) for 2-year-old toddlers, filled out by their parents, for a direct assessment of their linguistic skills. Any dimension at/or below the 10th percentile has been identified as language delay.

### Stimuli paradigm

A well-known speech prosody discrimination paradigm was used [20–22]. A female speaker using infant-direct speech was reading the children’s picture book *A Hungry Snake*, and the complete speech samples were collected. The speech samples were edited by Audacity editing software into five 15-second forward speech segments with well-formed prosodic units and the reverse (time) was selected to invert each speech segment into backward speech segments with the same phonetic features but with distorted semantic and prosodic information. Experimental paradigm editing was performed using E-Prime 3.0 (PST, USA). The presentation of forward and backward speech segments was counterbalanced and presented in a computer-generated pseudorandom order, and each was followed by a randomized 10–15 s. During the presentation of the stimuli, a video of the stationary silent, non-social stimuli was played on the computer screen in order to maintain the child’s attention and minimize motion artifacts as prior design [23–25] for a duration of 4 min.

### fNIRS collection

**Fig. 2:**
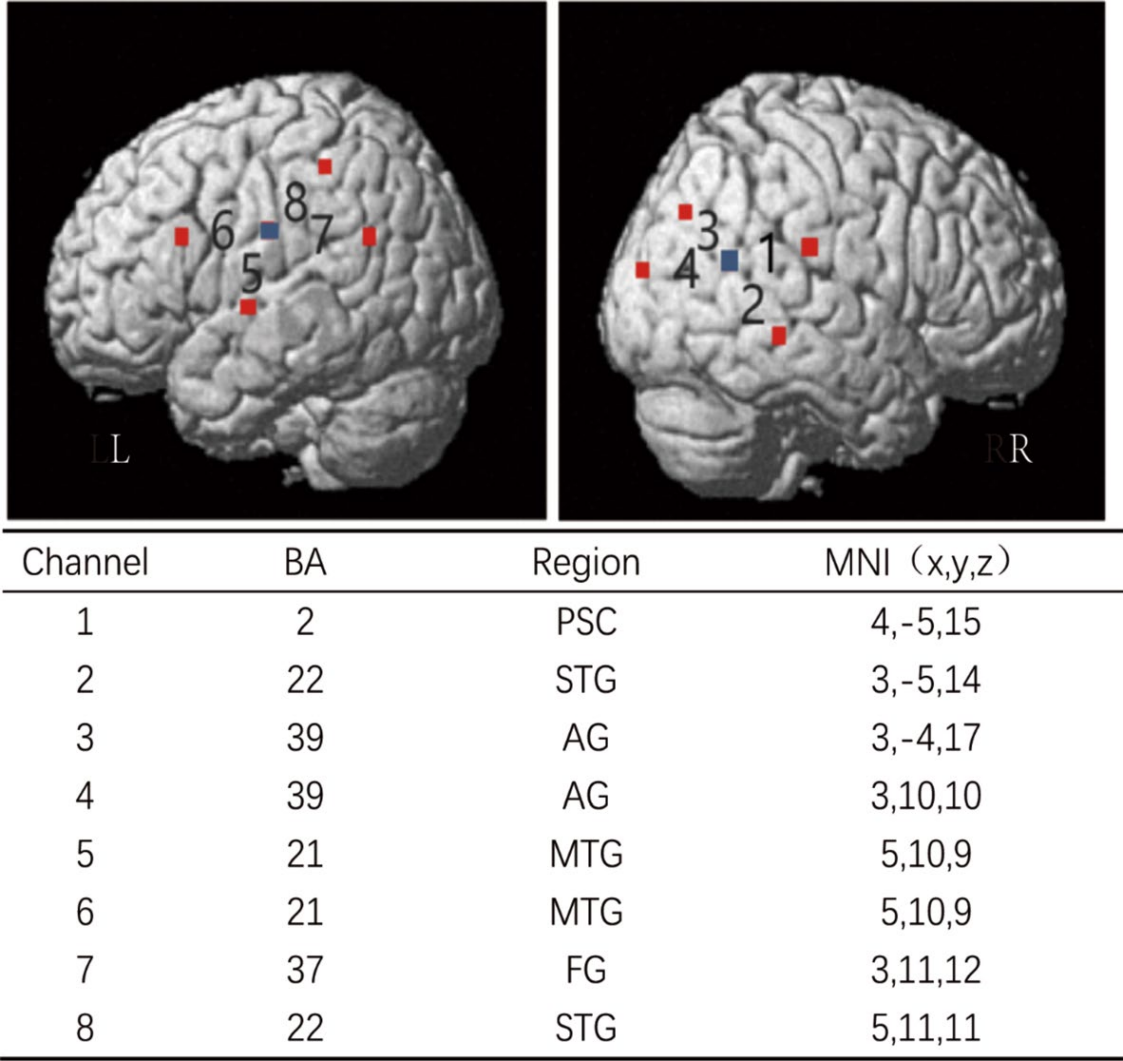
fNIRS channel and probe configuration. The 3D map illustrated the distribution of probes (red and blue representing the source and detector probe, respectively) and 8 channels. Note: L: the left brain; R, the right brain; BA, Brodmann area; MNI, montreal neurological institute coordinate; PSC, primary somatosensory cortex; STG, superior temporal gyrus; AG, angular gyrus; MTG, middle temporal gyrus; FG, fusiform gyrus

Data were recorded using a multiple-channel fNIRS system (Oxymon+, Artinis, Netherland). The separation between emitters and detectors was 3 mm. Two different wavelengths in the near-infrared range (760 nm and 850 nm, respectively) were used to measure the changes in optical density. The differential path length factor constant was set to 5.66, and the sampling rate was set at 10 Hz for data acquisition. Each pad comprising 4 channels was located directly above the ear (channels 1, 2, 3, and 4 in the right hemisphere, channels 5, 6, 7, and 8 in the left hemisphere) with the temporal areas, which have been shown to be voice sensitive in previous research in children and adults (Fig. 2). Toddlers were tested in a quiet lit room. The position of the head was supported with a gauze diaper to ensure a straight posture of the head and neck. One parent attended the measurement. The stimuli were presented using two loudspeakers positioned at a distance of approximately 60 cm in front of the toddler.

fNIRS data were pre-processed using open-source software homr2_UI, which is implemented in MATLAB (Mathworks, Natick, MA) [26]. Firstly, raw optical density data was converted to optical density units; motion artifact detection parameter settings: tMotion = 0.5 s; tMask = 1 s; standard deviation threshold = 50; amplitude threshold = 5. Then, motion artifacts were filtered out using a principal component analysis to correct motion artifacts by filtering out components that accounted for 99% of the data covariance. There with the high-pass filter was set to 0.01 to eliminate activity disturbances such as heart rate and respiration, and the low-pass filtered with a cutoff frequency of 0.5 Hz to eliminate high-frequency instrumental noise from the optical density data. The HbO and HbR concentration changes were then calculated using the modified Beer-Lambert law, with the differential path factor for the two wavelengths set to 6.0, and the motion artifact correction was set to 1.

We focused especially on HbO, as this variable has been reported to be the strongest marker of neural responses in children fNIRS [27]. Considering the lag in hemodynamic response, in each of the 8 channels, the average block started from 5 to 20 s post-stimulus onset for forward speech stimuli and backward speech stimuli, and 5 s pre-stimulus onset to onset for silence baseline. HbO changes of different speech stimuli was used to subtract HbO caused by a silent stimulus to obtain the HbO change value of the corresponding speech condition. For further analysis, prosody discrimination abilities were assessed by calculating the difference between HbO changes following forward speech stimuli and backward speech stimuli [22].

### Statistical analysis

A paired t-test was used to compare HbO changes following forward speech stimuli and backward speech stimuli in each channel, and the results were corrected using the multiple comparison Bonferroni method. Then, Pearson’s correlation coefficient was used for bivariate normally distributed data, and Spearman’s correlation coefficient was used for bivariate non-normally distributed or ranked data to analyze the correlation between the raw scores of the CCDI scale and the ability of each channel to discriminate rhythms. Furthermore, multifactorial logistic regression analyses were performed with the categorical variables sex and age as control variables, the continuous variable prosody discrimination ability of channels 1 to 8 as independent variable, and the outcome of language delay as dependent variable. The level of significance was a two-sided *P* value < 0.05. All analyses were performed in the SPSS26.0 software (IBM Corporation, Armonk, NY, USA).

Based on the bootstrap aggregation method and decision tree unit, the random forest (RF) model selects several new datasets by sampling the original dataset with put-back and trains the classifiers, then classifies the new samples by the ensemble of classifiers, and then statistically counts the classifications of all the classifiers by either majority voting or averaging over the outputs to provide a comprehensive prediction and classification. The clinical prediction model examines the relationship between neural prosody discrimination ability and language abilities in toddlers, aiming to predict language development outcomes based on early auditory processing capabilities. The RF model was chosen for its robustness in handling complex, nonlinear relationships and its ability to manage overfitting, making it highly effective for predicting outcomes from diverse and multidimensional data such as auditory processing and language ability metrics [28].

The RF was implemented using R. version 4.0.2 (R Core Team 2014) using the “random forest” package, to rank the factors that are significantly related to speech delay in preterm toddlers, to identify the relatively important factors that affect the speech development of preterm toddlers, and to establish a prediction model for the occurrence of speech delay in preterm toddlers. The data set adopted 10-fold cross validation. The graphical receiver operating characteristic (ROC) curve is produced, and the area under the ROC curve (AUC) is a performance index to measure the effectiveness of the model.

## Results

### The *phase I* study

#### General clinical characteristics of participants

Of the 98 full-term toddlers in the *phase I* study, 50 toddlers aged 1- year old and 48 toddlers aged 2- years-old, GA (38.95 ± 1.10) [37 to 41] weeks, birth weight (3199.18 ± 416.26) [2350, 4060] grams. In 1-year-old term toddlers (50 toddlers, 26 boys/24 girls, 12.1 ± 0.4 months old), CCDI vocabulary and gesture showed that the early gesture score was 17.58 ± 5.32, the later gesture score was 14.22 ± 6.68, the total gesture score was 31.8 ± 11.05, and the phrase understanding score was 20.88 ± 4.86. CCDI vocabulary and sentence results showed that the score of vocabulary comprehension was 219.78 ± 95.35, and the score of vocabulary expression was 5.3 ± 5.5. In 2-year-old term toddlers (48 toddlers, 28 boys/20 girls, 24.1 ± 0.9 months old), the vocabulary expression score was 625.33 ± 157.75, the sentence complexity score was 63 ± 25.44. The total screening abnormality rate was 9.2%, and there was no significant difference between different genders (*χ*2 = 0.536, *P*<0.05). The screening abnormality rates of participants included and not included in the *phase I* study were not significantly different.

#### Characteristic of brain prosody discrimination ability in full-term toddlers

**Fig. 3:**
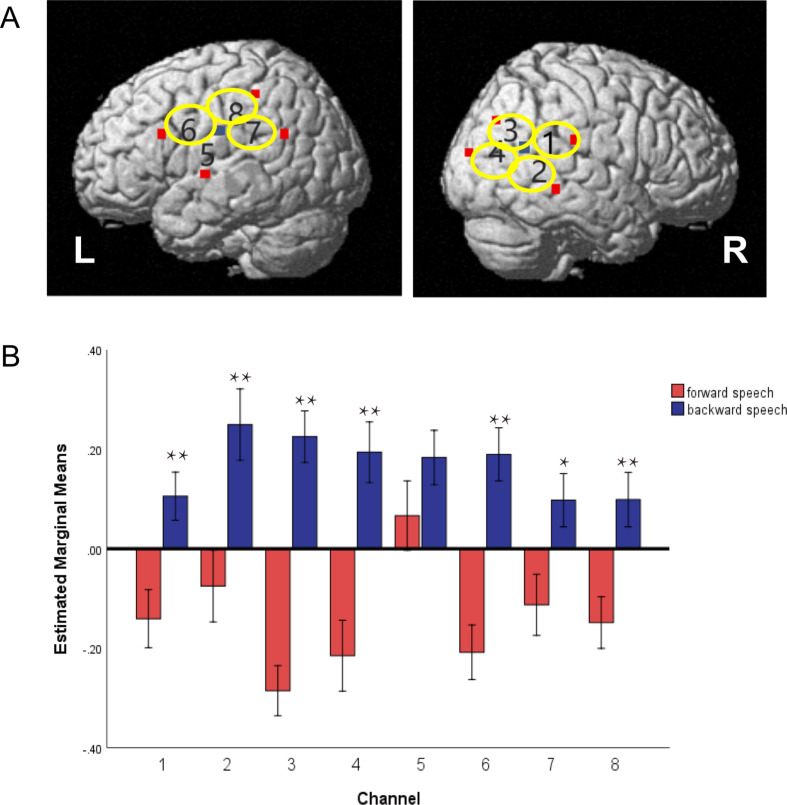
Brain prosody discrimination abilities in full-term toddlers (**A**) Map of activation areas in the left and right brain regions in full-term toddlers; (**B**) Bar charts show the difference in brain activation between forward speech and backward speech conditions. Note: *, *P* < 0.05, **, *P* < 0.01

Compared with stimulation of forward speech with good prosody, the stimulation of reverse speech without good prosody induced higher HbO response, including right primary sensory cortex channel 1, right superior temporal gyrus channel 2, right angular gyrus channel 3, right angular gyrus channel 4, left middle temporal gyrus channel 6, left fusiform gyrus channel 7, and left superior temporal gyrus channel 8 (*P* < 0.05). There was no significant difference in the changes of brain hemoglobin in other channels (Fig. 3).

#### The relationship between brain prosody discrimination ability and language development

In 1-year-old toddlers, the verbal prosody discrimination of channel 3 (angular gyrus) was correlated with the early gesture score. Total gesture score and total word comprehension score in CCDI vocabulary and gesture were significantly correlated with each other (*r* = 0.326 ～ 0.409, *P* < 0.05), and there was no significant correlation between the remaining channels and CCDI vocabulary and gesture scores (see Supplementary Table S1, Additional File S1). In 2-year-old toddlers, the verbal prosody discrimination of channel 3 (angular gyrus) was significantly positively correlated with the scores of CCDI vocabulary and sentence expression and sentence complexity (*r* = 0.331 ～ 0.387, *P* < 0.05). There was a significant positive correlation between the prosody discrimination of channel 4 (angular gyrus) and the sentence complexity score (*r* = 0.285, *P* < 0.05), while there was no significant correlation between the other channels and the scores of CCDI words and sentences (See Supplementary Table S2, Additional File S1). The prosody discrimination of channel 3 (right angular gyrus) in toddlers with language delay was significantly lower than that in toddlers with normal language development (*P* < 0.01), and there was no statistically significant difference between the other two aspects (see Supplementary Fig. S1, Additional File S1). Prosody discrimination of the right angular gyrus (channel 3) had a statistically significant effect on language delay (OR = 0.301, 95%CI: 0.092–0.984, *P* < 0.05) (Table 1).

**Table 1:** Multifactorial logistic analysis of language delay in full-term toddlers

	*β*	S.E.	Wald	Odd ratio	Lower	Upper	*P*
1BA	0.050	0.536	0.009	1.051	0.368	3.005	0.926
2BA	-0.350	0.461	0.577	0.705	0.285	1.740	0.448
3BA	-1.202	0.605	3.944	0.301	0.092	0.984	0.047*
4BA	0.517	0.486	1.131	1.677	0.647	4.352	0.288
5BA	-0.097	0.589	0.027	0.907	0.286	2.879	0.869
6BA	-0.015	0.647	0.001	0.985	0.277	3.499	0.981
7BA	0.428	0.744	0.330	1.534	0.357	6.596	0.566
8BA	-0.962	0.682	1.986	0.382	0.100	1.456	0.159

*Note:* *, *P* < 0.05, **, *P* < 0.01

### The *phase II* study

#### General clinical characteristics of participants

Of the 108 preterm toddlers in *phase II* study, 51 children aged 1-year old and 57 children aged 2-years old, GA (32.01 ± 2.99) [25 to 36] weeks, birth weight (1787.57 ± 588.25) [780, 3259] grams. In 1-year-old preterm toddlers (51 toddlers, 31 boys/20 girls, 11.9 ± 0.6 months old), the results of CCDI vocabulary and gesture showed that the early gesture score was 15.59 ± 4.39, the later gesture score was 12.25 ± 6.86, the total gesture score was 27.84 ± 9.58, and the phrase understanding score was 17.63 ± 5.69. The score of vocabulary comprehension was 160.49 ± 104.04, and the score of vocabulary expression was 3.47 ± 6.01. In 2-year-old preterm toddlers(57 toddlers, 31 boys/26 girls, 24.34 ± 0.4 months old), the score of vocabulary expression was 432.58 ± 267.67, the score of sentence complexity was 38.42 ± 26.54. The total screening abnormality rate was 34.3%, and there was no statistical difference between different genders(*χ*2 = 0.006, *P*<0.05). The screening abnormality rates of participants included and not included in the *phase II* study were not significantly different. Characteristics of preterm toddlers are shown in Supplementary Table S3, Additional File S1.

#### Characteristic of brain prosody discrimination ability in preterm toddlers

**Fig. 4:**
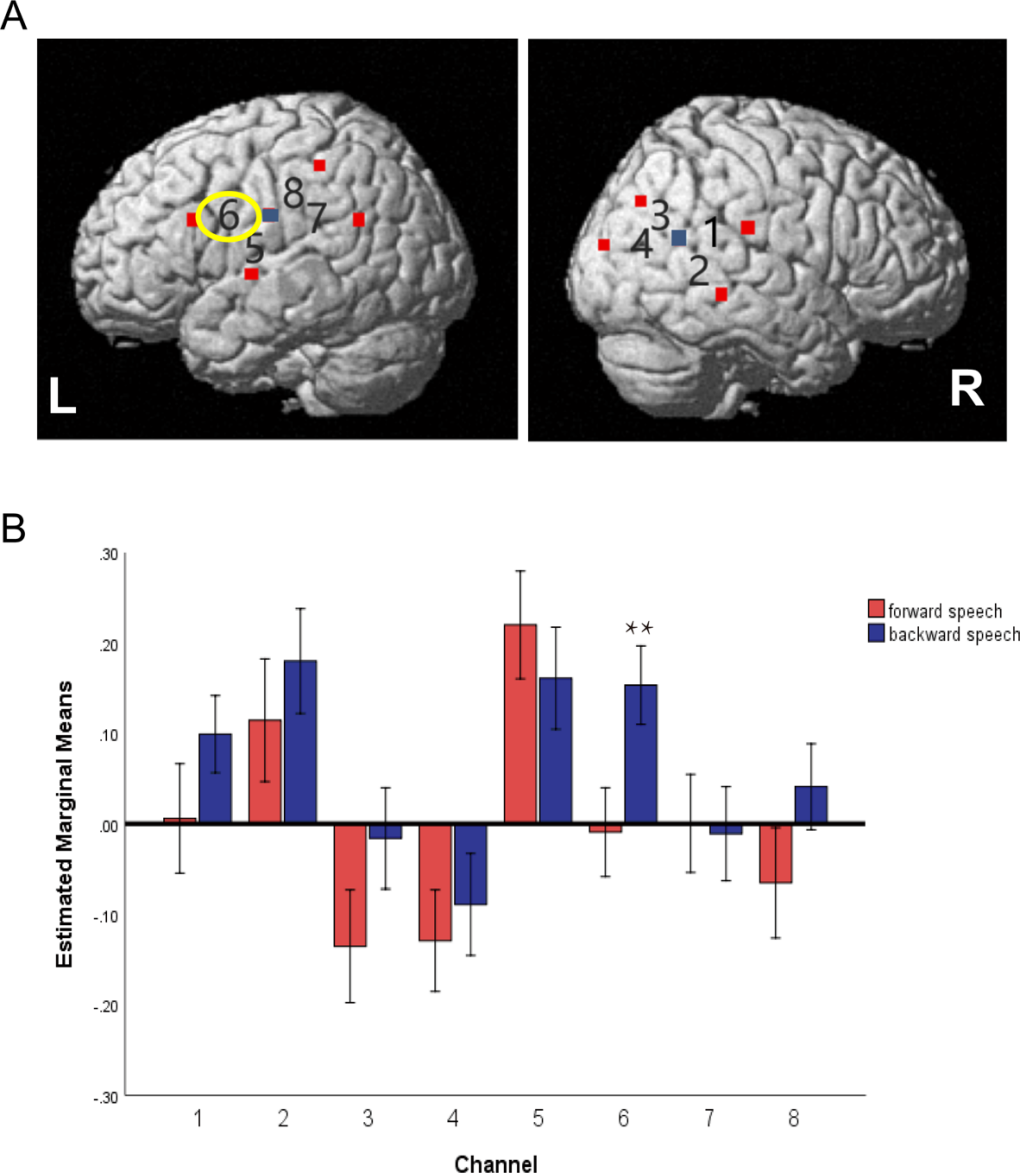
Brain prosody discrimination abilities in preterm toddlers (**A**) Map of activation areas in the left and right brain regions in preterm toddlers; (**B**) Bar charts show the difference in brain activation between forward speech and backward speech conditions. Note: *, *P* < 0.05, **, *P* < 0.01

Characteristic of brain prosody discrimination ability in preterm toddlers. In channel 6 (left middle temporal gyrus), compared with the forward speech condition, the brain activation of the reverse speech was significantly increased (*P* < 0.01), while in the other channels, there was no statistical difference in the changes of brain hemoglobin between the two kinds of speech, suggesting that only the left middle temporal gyrus channel 6 had a significant prosody discrimination (Fig. 4).

#### The relationship between brain prosody discrimination ability and language development

In 1-year-old toddlers, there was a significant correlation between the verbal prosody discrimination of channel 3 (right angular gyrus) and the understanding of CCDI vocabulary and gesture vocabulary (*r* = 0.454, *P* < 0.01), no significant correlation was found between the prosody discrimination of the residual channel and the dimensions of CCDI vocabulary and gesture (see Supplementary Table S4, Additional File S1). In 2-year-old toddlers, the verbal prosody discrimination of channel 3 (right angular gyrus), channel 4 (right angular gyrus), and channel 6 (left middle temporal gyrus) were significantly positive correlated with the scores of CCDI vocabulary and sentence expression and sentence complexity (*r* = 0.269 ~ 0.374, *P* = 0.005 ~ 0.043), no significant correlation was found between the prosody discrimination of other channels and the scores of CCDI words and sentences (see Supplementary Table S5, Additional File S1).

Furthermore, speech prosody discrimination of toddlers with language delay in channel 3 (right angular gyrus), channel 4 (right angular gyrus), and channel 6 was significantly lower than that of toddlers with normal language development (*P* < 0.05), but there was no significant difference for other channels (see Supplementary Fig. S2, Additional File S1).

#### Construction of prediction model for language delay in preterm toddlers

**Fig. 5:**
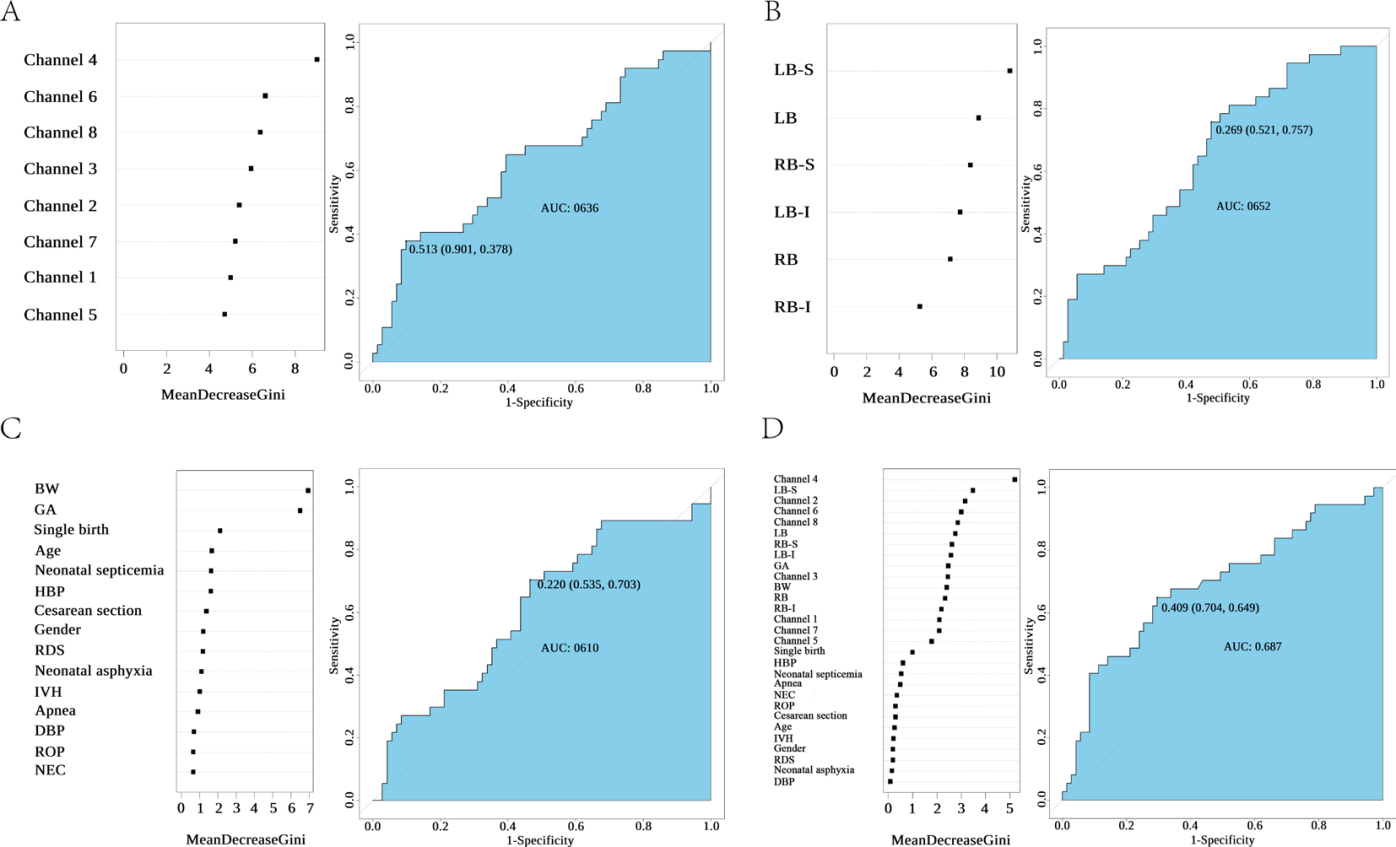
Random Forest modeling of language delay (**A**) Variables of each channel’s prosody discrimination abilities and random forest modeling of language delay (AUC = 0.636); (**B**) Variables of each brain area prosody discrimination abilities and random forest modeling of language delay (AUC = 0.652); (**C**) Variables of births and postnatal complications and random forest modeling of language delay (AUC = 0.610); (**D**) Combine the above variables and random forest modeling of language delay (AUC = 0.687). Note: LB, left brain region; RB, right brain region; S: superior; I: inferior; GA: gestational age; BW, birth weight; IVH, intra-ventricular hemorrhage; RDS, respiratory distress syndrome; BDP, bronchopulmonary dysplasia, NEC, neonatal necrotizing enterocolitis; ROP, retinopathy of prematurity; HBP: Hyperbilirubinemia with phototherapy; AUC: area under receiver operating characteristic

A total of four RF was constructed in this study to predict language delay. Firstly, the prosody discrimination of 8 channels was incorporated into RF, and the results showed that the prosody discrimination on channel 4 was the most important in predicting the development of language delay in preterm toddlers, and the area under the ROC curve was 0.636 (Fig. 5A). Secondly, based on the baseline data of channels 1 to 8, a random forest model was constructed to reflect the parameter combination of prosody discrimination of each brain region, suggesting that the upper left-brain region (combined by left superior temporal gyrus and middle temporal gyrus) was the most important brain region for predicting language delay in premature toddlers, and the area under the ROC curve of the model was 0.652 (Fig. 5B). Thirdly, some baseline data was incorporated into the random forest model together with postnatal complications, and it was found that birth weight and gestational age were important factors in predicting language delay in preterm toddlers, the area under the ROC curve was 0.610 (Fig. 5C). Lastly, based on the above parameters, a random forest model was constructed, and the results showed that the prosody discrimination reflected by channels and brain regions based on fNIRS data was an important parameter for predicting language delay in preterm toddlers, among which the prosody discrimination reflected by channel 4 was the most important parameter. The area under the model ROC curve was 0.687 (Fig. 5D).

## Discussion

This study shows that the prosody discrimination ability of children’s brains is positively associated with language development, specifically: (1) Neural prosody discrimination ability in the AG area showed a positive relationship with gesture and vocabulary compression, vocabulary expression, and sentence complexity. (2) Compared with full-term toddlers, preterm toddlers had decreased prosodic discrimination in multiple channel brain regions. Furthermore, we confirmed this relationship in RF, and the result showed that the deficit of prosodic discrimination in AG may be the main reason for worse language development in preterm toddlers.

### Prosody discrimination abilities play an important role in the language development

Prosodic features can provide information about the structure of speech sounds, and prosodic changes may help infants adapt to speech sounds, giving them the opportunity to recognize, analyze, and segment words and phrases during language acquisition [12, 29]. Given that, the prosodic cues provide useful information about the structure of language, prosodic discrimination may be a key foundational skill for language acquisition. This link between prosodic discrimination and language development, suggests that prosodic discrimination difficulties may increase the risk of delayed language acquisition.

This study showed that neural prosody discrimination was positively associated with language development in full-term toddlers. Previous studies have shown that infants rely on prosody to distinguish between languages and to detect differences in boundaries in speech, tonal contours of words, or patterns of lexical stress [30, 31]. Usha Goswami et al. evaluated the discrimination of children’s speech prosody and speech amplitude changes through the amplitude modulation pattern of electroencephalogram, and the study showed that the impairment of prosodic pattern was related to developmental language disorders, suggesting that the discrimination of prosodic information is the core of language acquisition [32]. Therefore, brain prosody discrimination is closely related to language development, and the evaluation of the brain prosody discrimination ability is of great significance to evaluate the level of children’s language development.

### Deficit of prosody discrimination abilities in right AG may the main reason for language delay

Furthermore, our results showed that the prosody discrimination of right AG in full-term toddlers with language delay was significantly lower than that of toddlers with normal language development. As well as, multivariate logistic regression analysis of the predictive effect of 8 channels on the outcome of language delay showed that the higher the prosody discrimination abilities, the lower the risk of language delay. Previous behavioral research used the head turn preference procedure of short phrase prosody to evaluate prosody discrimination ability and found that behavioral indicators of prosody sensitivity of 6-month-old infants could be used to predict vocabulary size after 18 months [33]. In addition, the ability to process phrase prosody also affects important aspects of learning in the later stages of language development, including information organization, word segmentation, and syntactic analysis in conversation [34]. Therefore, the assessment of early neural prosody discrimination development based on fNIRS plays an important role in the early detection of language delay.

### Construction of prediction model for language delay in preterm toddlers

Moreover, we confirmed this relationship in RF, which showed that a deficit of prosodic discrimination in AG may be the main reason for worse language development in preterm toddlers. In our study, the RF model constructed for the prosody discrimination ability of channels 1 to 8, the result showed that channel 4 in the right AG was relatively most important predictor of language delay. RF models constructed for each brain region showed that prosody discrimination ability in the left supratentorial region, comprising left MTG channel 6 and left STG channel 8 was the most important predictor of outcome. RF models constructed for baseline and postnatal complications showed that birth weight and gestational age were the most important predictors of outcome, consistent with a previous review study [11]. RF was constructed by combining all the parameters, suggesting that the prosody discrimination ability reflected by each channel and brain region based on the fNIRS data was an important parameter in predicting preterm toddlers with language delay, and right AG channel 4 being the most highly weighted, followed by left MTG channel 6, with an area under the ROC curve of 0.687, suggesting these parameters have fair accuracy as the predictor variables in predicting the occurrence of language delay in preterm toddlers.

Compared to full-term toddlers, preterm toddlers showed a much higher screening abnormality rate and prosody discrimination ability in the left MTG only (channel 6), while full-term toddlers showed in all channels except channel 5, suggesting that preterm toddlers have decreased prosodic discrimination in multiple channel brain regions. In previous studies, the fNIRS results of verbal prosody discrimination of sleeping infants showed that the forward speech extending from the upper temporal to the sub-parietal and the middle and lower frontal regions in the left hemisphere of the full-term infants showed significantly greater hemodynamic response than the reverse speech, but no difference in the hemodynamic response between forward and reverse speech in the preterm group, suggesting that preterm infants have deficit in prosody discrimination [35]. Likeness, fNIRS study on the speech prosody discrimination of newborns aged 23 to 41 weeks during sleep was also conducted, and the results showed that preterm infants with smaller gestational age are more likely to have deficit in early prosody discrimination [22]. Therefore, preterm birth may lead to prosody discrimination deficit in preterm children, and it could be a main mechanism for higher risk for language delay.

### Possible causes of prosodic development deficit in preterm children

Premature birth could lead to prosodic discrimination deficit in preterm children due to several reasons. First, previous studies have shown that auditory cortex maturation is interrupted by preterm birth [36], because the last three months of pregnancy is an important period for the development of the auditory cortex [37, 38, 39]. Second, most premature babies are monitored in neonatal care units after birth, during which they are deprived of the biological maternal sounds they hear in utero, including low-frequency sounds, the mother’s heartbeat, and intestinal sounds [40]. Third, children who breastfeed in incubators are exposed to high levels of noise caused by the air supply system, impeding their discrimination of speech coming from outside the incubator [35]. Furthermore, preterm toddlers often have low birth weight and birth complications that increase the risk of abnormal brain function [41, 42].Thus, the combination of these factors can lead to deficits in prosody discrimination in preterm children, and can ultimately lead to delayed language development.

Therefore, we need to pay special attention to the development of prosody discrimination, especially for preterm toddlers. fNIRS assessment, as an objective index for early identification of language delay, and can provide objective and reliable imaging indexes for the early identification of language delay. In addition, more attention should focus on toddlers with deficit prosody discrimination in the right AG area in the fNIRS assessment, and the trajectory of changes in the language development of these preterm infants should be dynamically monitored.

### Limitations and prospects

There are still some shortcomings in this study. As a single-center study, the sample size included was small, and the random forest yielded an area under the ROC curve of 0.687, which was a general ability to identify the outcome. However, this study is a first and preliminary analysis of the risk factors for the occurrence of language delay in preterm toddlers based on fNIRS, as well as an exploration and attempt to establish a prediction model for language delay in preterm children. In the future, our group plans to make a follow-up to this group of children and conduct a multi-temporal assessment to monitor the trajectory of language development in a long-term and dynamic manner. Furthermore, our group intends to conduct a multi-center study that encompasses a broader demographic and geographic population to improve the applicability of our findings.

fNIRS technology offers the potential for earlier detection of language delays in children than traditional methods. Its non-invasive nature and sensitivity to cortical hemodynamic responses make it suitable for use with infants and young children, enabling early intervention and potentially improving long-term language outcomes. But using fNIRS technology requires specialized knowledge for setup, operation, and interpretation of results, developing standardized protocols and guidelines for the use of fNIRS in language delay diagnosis and intervention could help to mitigate these challenges.

## Conclusions

The prosody discrimination of preterm toddlers was less developed than that of full-term toddlers, and it may be the reason for a higher screening abnormality rate. Neural prosody discrimination ability is positively associated with language development, assessment of brain prosody discrimination abilities through fNIRS can be used as an objective indicator for early identification of children with language delay in the future clinical application.

### Electronic supplementary material

Below is the link to the electronic supplementary material.

Supplementary Material 1

## Data Availability

The datasets used and/or analyzed during the current study are available from the corresponding author on reasonable request.

## Abbreviations

AG: Angular gyrus
AUC: Area under receiver operating characteristic
BA: Brodmann’s areas
BDP: Bronchopulmonary dysplasia
BW: Birth weight
CCDI: Chinese communicative development inventory mandarin version
FG: Fusiform gyrus
fNIRS: Functional near-infrared spectroscopy
GA: Gestational age
HbO: Oxy-hemoglobin
HbR: Deoxy-hemoglobin
HBP: Hyperbilirubinemia with phototherapy
IVH: Intra-ventricular hemorrhage
LB: Left brain region
LB-I: Left inferior brain region
LB-S: Left superior brain region
MTG: Middle temporal gyrus
MNI: Montreal neurological institute
NEC: Neonatal necrotizing enterocolitis
PSC: Primary somatosensory cortex
RB: Right brain region
RB-I: Right inferior brain region
RB-S: Right superior brain region
RDS: Respiratory distress syndrome
RF: Random forest
ROC: Receiver operating characteristic
ROP: Retinopathy of prematurity
STG: Superior temporal gyrus
