# Supplementary material for: Association between neural prosody discrimination and language abilities in toddlers: a functional near-infrared spectroscopy study
Source: BMC Pediatr. 2024 Jul 12;24:449. doi: 10.1186/s12887-024-04889-7 (PMC11241962; doi:10.1186/s12887-024-04889-7)
Supplement: Supplementary file 1 — Supplementary Material 1 [file 12887_2024_4889_MOESM1_ESM.docx]

## Additional file 1

**Supplementary Table 1** The associations between CCDI scores and prosody discrimination abilities in 50 full-term toddlers of 1-year-old

| Dimensions of CCDI | 1BA | 2BA | 3BA | 4BA | 5BA | 6BA | 7BA | 8BA |
| --- | --- | --- | --- | --- | --- | --- | --- | --- |
| Early gesture | 0.094 | -0.008 | 0.409** | -0.003 | -0.104 | 0.141 | -0.121 | -0.071 |
| Late gesture | -0.057 | -0.172 | 0.214 | -0.004 | -0.215 | -0.036 | -0.053 | -0.008 |
| Total gesture | 0.011 | -0.108 | 0.326* | -0.004 | -0.180 | 0.046 | -0.090 | -0.039 |
| Phrase comprehension | -0.001 | 0.115 | 0.152 | -0.145 | -0.150 | -0.038 | -0.103 | -0.080 |
| Vocabulary comprehension | -0.041 | -0.043 | 0.331* | -0.052 | -0.114 | 0.175 | -0.089 | -0.024 |
| Vocabulary expression | -0.014 | 0.036 | 0.106 | 0.062 | 0.262 | 0.187 | 0.152 | 0.082 |

Note: 1 to 8 BA represent the prosodic discrimination abilities of channels 1 to 8 respectively; *, *P* < 0.05, **, *P* < 0.01.

**Supplementary Table 2** The associations between CCDI scores and prosody discrimination abilities in 48 full-term toddlers of 2-years-old

| Dimensions of CCDI | 1BA | 2BA | 3BA | 4BA | 5BA | 6BA | 7BA | 8BA |
| --- | --- | --- | --- | --- | --- | --- | --- | --- |
| Vocabulary expression | 0.127 | 0.144 | 0.419** | 0.128 | 0.139 | 0.157 | 0.124 | 0.156 |
| Sentence complexity | 0.066 | 0.078 | 0.387** | 0.285* | 0.170 | 0.162 | 0.235 | 0.142 |

Note: 1 to 8 BA represent the prosodic discrimination abilities of channels 1 to 8 respectively; *, *P* < 0.05, **, *P* < 0.01.

**Supplementary Figure 1** Comparison of prosody discrimination between full-term toddlers with normal language and language delay


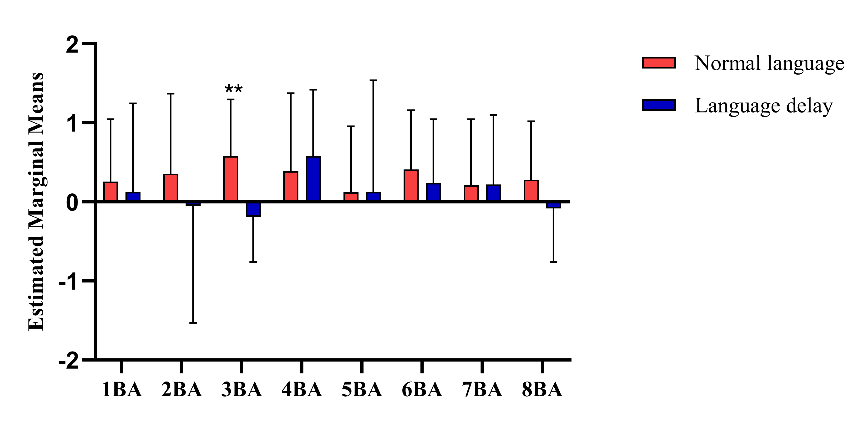


Note: 1 to 8 BA represent the prosodic discrimination abilities of channels 1 to 8 respectively; *, *P* < 0.05, **, *P* < 0.01.

**Supplementary Table 3** The characteristics of baseline, birth, and postnatal complications in 108 preterm toddlers

| Characteristic | *n* | categorical variable | Percentages |
| --- | --- | --- | --- |
| Sex | 62 | Male | 57.5% |
|  | 46 | Female | 42.6% |
| Age | 51 | 1-year-old | 47.2% |
|  | 57 | 2-years-old | 52.8% |
| Singleton | 26 | No | 24.1% |
|  | 82 | Yes | 75.9% |
| Cesarean section | 36 | No | 33.3% |
|  | 72 | Yes | 66.7% |
| Neonatal asphyxia | 96 | No | 88.9% |
|  | 12 | Yes | 11.1% |
| Hyperbilirubinemia with phototherapy | 42 | No | 38.9% |
|  | 66 | Yes | 61.1% |
| Intracranial hemorrhage | 45 | No | 41.7% |
|  | 63 | Yes | 58.3% |
| Respiratory distress syndrome | 51 | No | 47.2% |
|  | 57 | Yes | 52.8% |
| Neonatal apnea | 96 | No | 88.9% |
|  | 12 | Yes | 11.1% |
| Bronchopulmonary dysplasia | 95 | No | 88.0% |
|  | 13 | Yes | 12.0% |
| Necrotizing enterocolitis | 101 | No | 93.5% |
|  | 7 | Yes | 6.5% |
| Neonatal sepsis | 92 | No | 85.2% |
|  | 16 | Yes | 14.8% |
| Retinopathy of prematurity | 104 | No | 96.3% |
|  | 4 | Yes | 3.7% |

**Supplementary Table 4** The associations between CCDI scores and prosody discrimination abilities in 51 preterm toddlers of 1-year-old

| Dimensions of CCDI | 1BA | 2BA | 3BA | 4BA | 5BA | 6BA | 7BA | 8BA |
| --- | --- | --- | --- | --- | --- | --- | --- | --- |
| Early gesture | 0.064 | -0.028 | 0.020 | 0.096 | 0.194 | 0.208 | -0.070 | -0.005 |
| Late gesture | 0.125 | 0.040 | 0.269 | 0.043 | -0.119 | 0.173 | -0.202 | -0.083 |
| Total gesture | 0.119 | 0.016 | 0.202 | 0.075 | 0.004 | 0.219 | -0.176 | -0.062 |
| Phrase comprehension | 0.027 | -0.241 | 0.190 | 0.159 | 0.111 | 0.161 | -0.146 | 0.083 |
| Vocabulary comprehension | 0.099 | -0.167 | 0.454** | 0.271 | -0.041 | 0.213 | -0.167 | -0.180 |
| Vocabulary expression | -0.068 | 0.092 | 0.197 | 0.171 | -0.179 | 0.053 | 0.056 | 0.092 |

Note: 1 to 8 BA represent the prosodic discrimination abilities of channels 1 to 8 respectively; *, *P* < 0.05, **, *P* < 0.01.

**Supplementary Table 5** The associations between CCDI scores and prosody discrimination abilities in 57 preterm toddlers of 2-year-old

| Dimensions of CCDI | 1BA | 2BA | 3BA | 4BA | 5BA | 6BA | 7BA | 8BA |
| --- | --- | --- | --- | --- | --- | --- | --- | --- |
| Vocabulary expression | 0.098 | 0.070 | 0.370** | 0.344** | -0.042 | 0.303* | 0.091 | 0.224 |
| Sentence complexity | 0.039 | 0.021 | 0.374** | 0.347** | -0.060 | 0.269* | 0.098 | 0.191 |

Note: 1 to 8 BA represent the prosodic discrimination abilities of channels 1 to 8 respectively; *, *P* < 0.05, **, *P* < 0.01.

**Supplementary Figure 2** Comparison of prosody discrimination between preterm toddlers with normal language and language delay


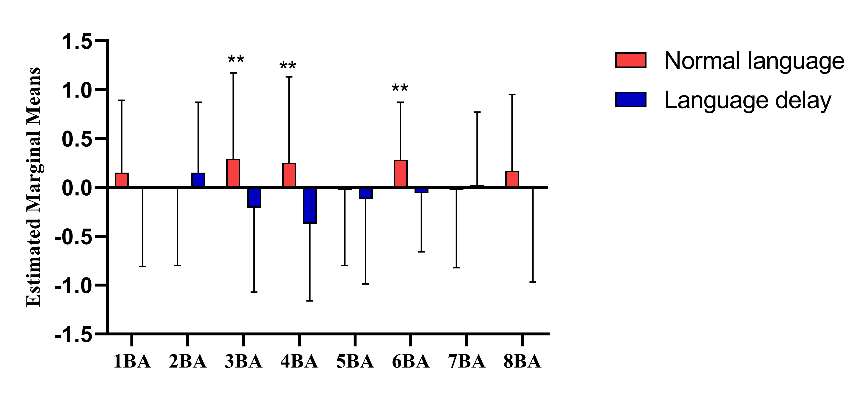


Note: 1 to 8 BA represent the prosodic discrimination abilities of channels 1 to 8 respectively; *, *P* < 0.05, **, *P* < 0.01.
